# Novel Optineurin Frameshift Insertion in a Family With Frontotemporal Dementia and Parkinsonism Without Amyotrophic Lateral Sclerosis

**DOI:** 10.3389/fneur.2021.645913

**Published:** 2021-05-19

**Authors:** Jacqueline Dominguez, Jeryl Tan Yu, Yi Jayne Tan, Arlene Ng, Ma Fe De Guzman, Boots Natividad, Ma Luisa Daroy, Jemellee Cano, Justine Yu, Michelle M. Lian, Li Zeng, Weng Khong Lim, Jia Nee Foo, Adeline S. L. Ng

**Affiliations:** ^1^Institute for Neurosciences, St. Luke's Medical Center, Quezon City, Philippines; ^2^Department of Neurology, National Neuroscience Institute, Singapore, Singapore; ^3^Research and Biotechnology Division, St Luke's Medical Centre, Quezon, Philippines; ^4^Lee Kong Chian School of Medicine, Nanyang Technological University, Singapore, Singapore; ^5^Neural Stem Cell Research Lab, Research Department, National Neuroscience Institute, Singapore, Singapore; ^6^Neuroscience and Behavioural Disorders Programme, Duke-NUS Medical School, Singapore, Singapore; ^7^Singhealth Duke-NUS Institute of Precision Medicine, Singapore, Singapore; ^8^Cancer & Stem Cell Biology Program, Duke-NUS Medical School, Singapore, Singapore; ^9^Human Genetics, Genome Institute of Singapore, A*STAR, Singapore, Singapore

**Keywords:** familial FTD, novel mutation, parkinsonism, amyotrophic lateral scelerosis, optineurin *(OPTN)*

## Abstract

Frontotemporal Dementia (FTD) is a common cause of Young Onset Dementia and has diverse clinical manifestations involving behavior, executive function, language and motor function, including parkinsonism. Up to 50% of FTD patients report a positive family history, supporting a strong genetic basis, particularly in cases with both FTD and amyotrophic lateral sclerosis (FTD-ALS). Mutations in three genes are associated with the majority of familial FTD (fFTD) cases - microtubule associated protein tau gene (*MAPT*), granulin precursor (*GRN*), and hexanucleotide repeat expansions in chromosome 9 open reading frame 72- SMCR8complex subunit (C9orf72) while mutations in other genes such as optineurin (*OPTN*) have rarely been reported. Mutations in *OPTN* have been reported mostly in familial and sporadic cases of ALS, or in rare cases of FTD-ALS, but not in association with pure or predominant FTD and/or parkinsonian phenotype. Here, we report for the first time, a family from the Philippines with four members harboring a novel frameshift insertion at *OPTN* (Chr 10:13166090 G>GA) p.Lys328GluTer11, three of whom presented with FTD-related phenotypes. Additionally, one sibling heterozygous for the frameshift insertion had a predominantly parkinsonian phenotype resembling corticobasal syndrome, but it remains to be determined if this phenotype is related to the frameshift insertion. Notably, none of the affected members showed any evidence of motor neuron disease or ALS at the time of writing, both clinically and on electrophysiological testing, expanding the phenotypic spectrum of *OPTN* mutations. Close follow-up of mutation carriers for the development of new clinical features and wider investigation of additional family members with further genetic analyses will be conducted to investigate the possibility of other genetic modifiers in this family which could explain phenotypic heterogeneity.

## Introduction

Frontotemporal Dementia (FTD) is a common cause of Young Onset Dementia defined as dementia occurring before age 65, and has diverse clinical manifestations affecting behavior, executive function or speech (1, 2). The most common clinical phenotype remains behavioral variant FTD (bvFTD) (3), with two other language-predominant phenotypes i.e., non-fluent primary progressive aphasia (nfvPPA) and semantic variant primary progressive aphasia (svPPA) (4). Between 30–50% of patients report a family history of FTD in at least one family member, supporting a strong genetic basis (1). Mutations in three genes are associated with the majority of familial FTD (fFTD) cases - microtubule associated protein tau gene (*MAPT*), granulin precursor (*GRN*), and hexanucleotide repeat expansions in chromosome 9 open reading frame 72- SMCR8complex subunit (C9orf72) (5). C9orf72 repeat expansions are the most common genetic cause of ALS-FTD spectrum disorder (6, 7), but mutations in other genes have also been linked with familial ALS-FTD (8–11).

Mutations in optineurin (*OPTN*) have been identified mostly in familial and sporadic cases of amyotrophic lateral sclerosis (ALS) (12). *OPTN* mutations were first linked to ALS in the Japanese population, accounting for 3.3% of familial ALS (FALS) and 0.2% of sporadic ALS (sALS) patients (13). Rare reports of *OPTN* mutations linked to FTD or displaying an FTD phenotype include a Chinese patient heterozygous for a *OPTN* c.1546G> C (p.E516Q) mutation with a rapidly progressive ALS-FTD phenotype (14), and two male patients with onset in the 5th decade of ALS-FTD, carrying novel compound heterozygous loss-of-function mutations in *OPTN* (nonsense variant p.Ser262* and frameshift deletion p.Leu430Argfs*16) (15), and heterozygous for the *OPTN* p.E478G variant (16), respectively. A novel homozygous splice-site variant (c.1242þ1G>A) was recently reported in a patient with logopenic variant FTD (lvFTD) and whose sibling was diagnosed with non-fluent variant FTD. No ALS was reported in these siblings (17).

Here, we report for the first time, a novel frameshift insertion at *OPTN* (Chr 10:13166090 G>GA) p.Lys328GluTer11 in 4 members of a family from the Philippines. Affected members harboring homozygous mutation presented with FTD-related phenotypes. Notably, none of the affected members showed any evidence of motor neuron disease or ALS at the time of writing, both clinically and on electrophysiological testing.

## Materials and Methods

### Familial Investigation

Participants belonged to a family from Samar, region of the Visayas Islands in the Philippines, who presented to the Memory Center at St Luke's Medical Center with varying FTD phenotypes. Interviews with surviving family members were conducted. This study was approved by the St. Luke's Institutional Ethics Review Committee (Ethics Clearance IERC Ref. No. CT-14089). Written informed consent was obtained from all patients (or their caregivers) who participated in this study.

### Clinical Studies

All participants underwent standardized medical history and neurologic evaluations by specialist cognitive neurologists, as well as cognitive screening at the Memory Center, including the Mini Mental State Exam (MMSE) (18), Montreal Cognitive Assessment- Philippines (MoCA-P) (19), Clinical Dementia Rating (CDR) Assessment, comprehensive psychometric evaluation and motor evaluation using the Unified Parkinson's Disease Rating Scale (UPDRS) (20).

### Diagnostics and Neuroimaging Studies

Participants underwent cranial Magnetic Resonance Imaging (MRI)dementia protocol with hippocampal volumetry. Cranial PET-CT was performed using 2-F18-Fluoro-2-deoxy-D-glucose (^18^F-FDG). Electromyography and nerve conduction studies (EMG-NCV) and muscle ultrasound were done at the Neurophysiology Unit of the same hospital.

### Molecular Genetic Studies

Genomic DNA was extracted from peripheral blood from the proband and five siblings (two symptomatic, three asymptomatic). FTD-associated genes were sequenced as part of a custom targeted exome sequencing panel of 200 neuro-degenerative disease-related genes as previously described (21) (Supplementary Table 1). Exonic sequences of these 200 genes were captured with the NimbleGenSeqCap EZ choice <7 Mb (Roche) following the manufacturer's protocol. The captured samples were pooled and submitted as one lane of 151 bp paired-end sequencing with HiSeq4000 (Illumina). Sequence reads were aligned to the human reference genome (hg19) using BWA-MEM algorithm (BWA, v0.7.15) (22) and variants were called using Genome Analysis Tool-Kit (GATK, v3.7) according to GATK best practices (23–25). Variants were annotated using ANNOVAR (26), and filtered to retain only rare (gnomADMAF <5%) non-synonymous, frameshift, stop-gain and splice site variants (27–29). Mean coverage for the *OPTN* gene with targeted exome sequencing was 89.8x, with an average of 98.1% of *OPTN* targeted exons region (5,230 bp) covered by at least 15 reads (base quality ≥10, mapping quality ≥20). *OPTN* frameshift mutations were further confirmed by Sanger sequencing (Sanger and C9orf72 genotyping methods and primers used are described in Supplementary Material). Four family members with *OPTN* frameshift mutations were then sequenced by whole-exome sequencing (WES) to elucidate any further pathogenic mutation. Details on WES can be found in Supplementary Material. In the WES data, the mean coverage for the *OPTN* gene was 102.4x, with an average of 100% of *OPTN* targeted exons region (2,817bp) covered by at least 15 reads (base quality ≥10, mapping quality ≥20).

## Results

### Molecular Genetic Studies

**Figure 1:**
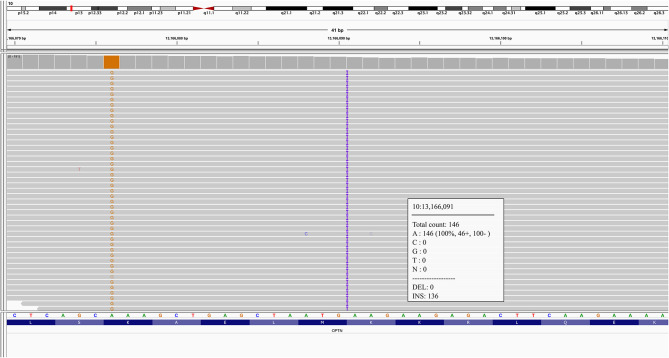
Integrative Genomics Viewer (IGV) inspection of proband III-10 at chr10:13166090, G>GA.

A novel frameshift insertion at *OPTN* (Chr 10:13166090 G>GA) p.Lys328 GluTer11 was identified in four family members (proband III-10 and two symptomatic siblings III-7 and III-8, and an asymptomatic sibling (III-6) by targeted exome sequencing (Figure 1). WES did not reveal any further pathogenic mutations. Siblings III-2 and III-9 were unaffected and did not carry the frameshift insertion. This insertion has not been seen in gnomAD (30). Genetic results, imaging findings, demographics, and clinical phenotypes are summarized in Table 1. The proband (III-10) and sibling III-8 were both homozygous for the frameshift insertion, while siblings III-7 and III-6 were heterozygous. No candidate mutations in the three most common FTD-related genes i.e., *MAPT, GRN*, and C9orf72 were found in any participant. In sibling III-7 with a parkinsonian phenotype, no pathogenic mutations in known PD-related genes were found.

**Table 1:** Summary of demographic, clinical and genetic profile of family members.

	**Age at onset/disease duration (years)**	**Sex**	**Phenotype**	**Gene mutation**	**MRI/PET**	**C9orf72 genotype**
III-10^**^	45/2	F	bvFTD	*OPTN* homozygous	MRI: bilateral temporal lobe atrophy	2,7
III-8	52/4	F	nfvPPA	*OPTN* homozygous	MRI: bilateral temporal lobe atrophy/Mild diffuse FDG hypometabolism in the left parietotemporal cortex	2,7
III-7	52/7	F	Parkinsonism	*OPTN* heterozygous	Unremarkable	6,6
III-6	^*^	F	^*^	*OPTN* heterozygous	Unremarkable	2,7
III-2	–	M	–	–	Unremarkable	6,6
III-9	–	F	–	–	Unremarkable	6,6

** *Proband;*

* *asymptomatic; III, Third generation; number next to III refers to birth order in the generation; bvFTD, behavioral variant frontotemporal dementia; nfvPPA, non-fluent variant primary progressive aphasia; OPTN, Optineurin gene*.

### Clinical Information

**Figure 2:**
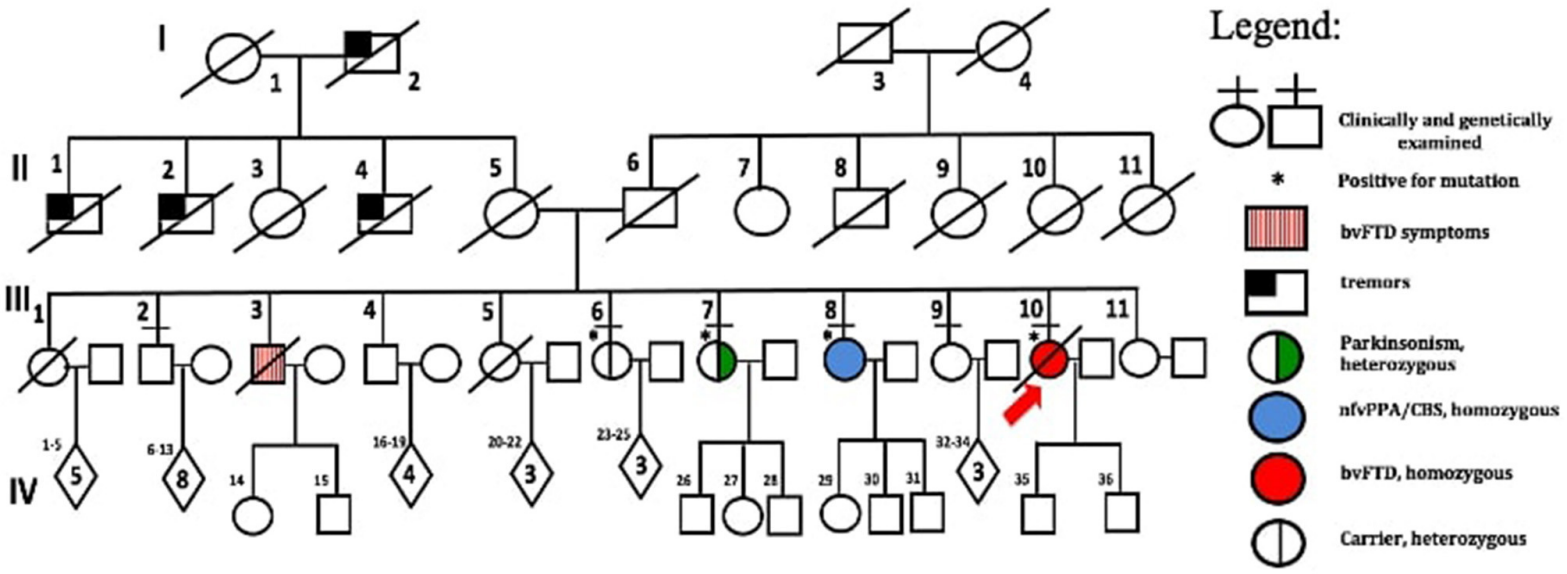
Family pedigree with generations and subjects labeled. Standard symbols were used.

**Figure 3:**
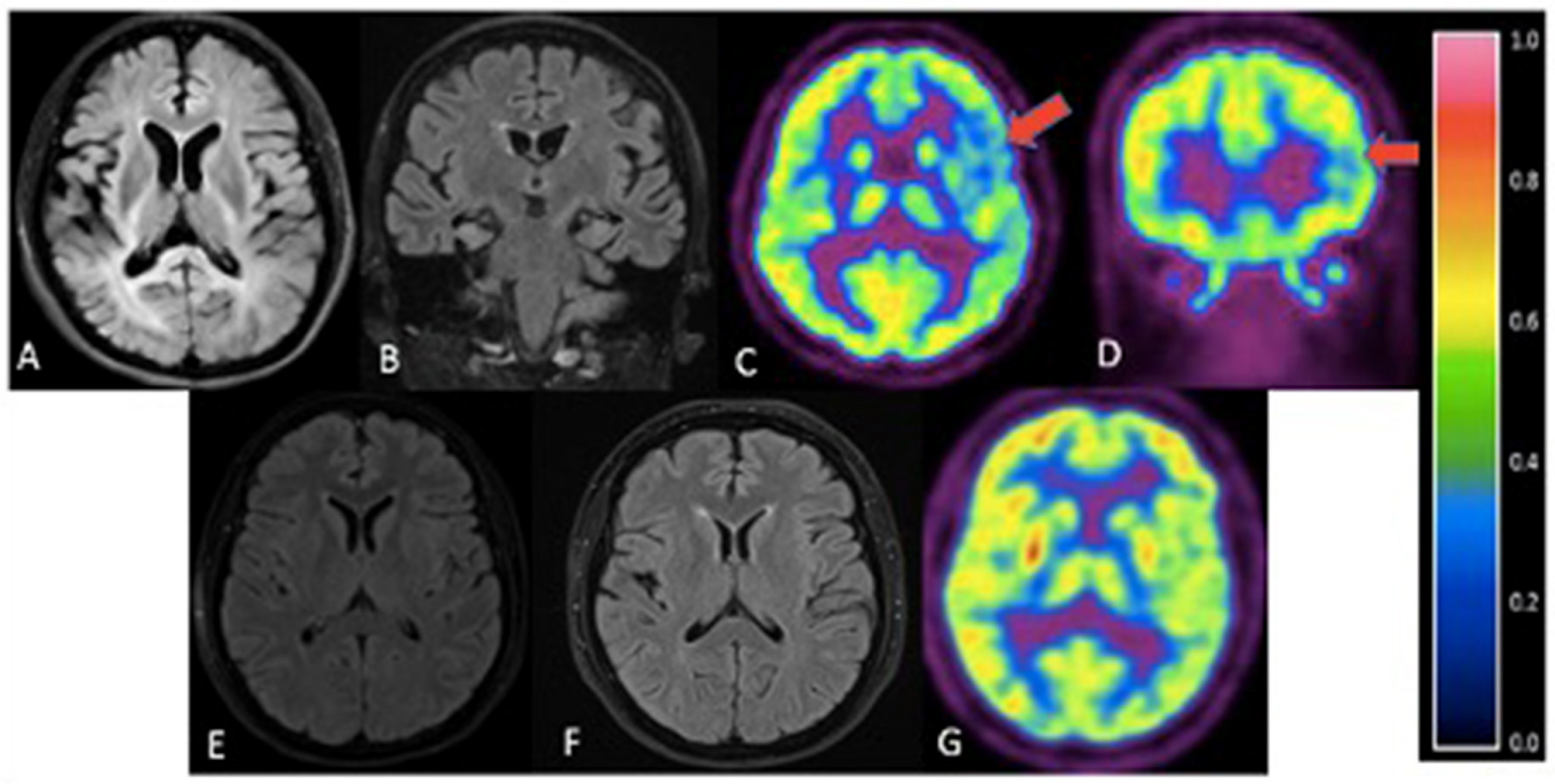
Cranial imaging of family members. **(A)** - III-10. Axial FLAIR. Mild atrophy of both temporal lobes. **(B)** - III-8. Coronal FLAIR MRI. Mild atrophy of bilateral parietotemporal areas. **(C,D)** III-8. Axial **(C)** and coronal **(D)** FDG-PET. Mild diffuse FDG hypometabolism in the left parietotemporal cortex (red arrows) with reference color scale to signify FDG uptake. **(E)** - III-6. Axial FLAIR. Unremarkable MRI. **(F)** - III-7. Axial FLAIR. No significant atrophy or hyperintensities. **(G)** - III-7. FDG-PET scan revealed no definite focus of abnormally increased or decreased FDG uptake.

The family consists of 62 members originally from Basey, Samar, Philippines, spanning four generations (Figure 2). Four individuals carried the *OPTN* frameshift mutation, of which three were symptomatic. This family belongs to category 2 based on the Goldman criteria for familial frontotemporal dementia. The proband, III-10 (Figure 2), presented with changes in behavior at 45 years of age, which included facing backwards while sitting as a pillion rider on the motorcycle, plucking her hair in bunches, and wandering around her village. Executive tasks became challenging and she was eventually home bound. She developed poor personal hygiene and lost interest in social interaction, eventually becoming no longer functional. In the clinic, she was garrulous and laughed inappropriately. At initial presentation, she had an MMSE score of 13/30 and a MoCA-P score of 4/30. Her comprehension was impaired, and she had difficulty following complex sequential commands. Cranial MRI showed mild atrophy of both temporal lobes (Figure 3A). She was clinically diagnosed with bvFTD but died 2 years after diagnosis at the age of 47.

Sibling III-8 presented with word-finding difficulty at age 52. She had frequent pauses and prolonged retrieval, but memory was preserved. She found it easier to express her thoughts via written instead of verbal means and frequently interchanged syllables during verbal expression. She also complained of stiffness of her upper back and left arm. The following year, she reported difficulty comprehending written text. There were no hallucinations or delusions. By 55 years of age, she was almost mute, communicating via gestures. There was decreased arm swing on the right; deep tendon reflexes were exaggerated in the right upper and lower extremities but there were no fasciculations, myoclonus, or dystonia. She had a positive snout reflex and positive Babinski sign bilaterally. She had a MoCA-P score of 23/30 and was clinically diagnosed with the non-fluent variant PPA with a corticobasal syndrome (CBS) phenotype. Serial follow-up showed progression of asymmetric parkinsonism. Cranial MRI showed bilateral temporal lobe atrophy (Figure 3B). FDG-PET showed mild diffuse FDG hypometabolism in the left parietotemporal cortex (Figures 3C,D). EMG and muscle ultrasound showed no fasciculations.

Sibling III-7 developed symptoms at 52 years of age with a parkinsonian phenotype consisting of bradykinesia and a stooped posture. Her language and cognition were intact, and she had no delusions, hallucinations, visual perceptual dysfunction or falls. She sought consult at our center 3 years after initial presentation of symptoms. She had a MoCA-P score of 27/30. Extraocular movements were full; she had a stooped posture with slight rigidity and asymmetric cogwheeling with tremors, more prominent on the right. She had a Unified Parkinson Disease Rating Scale (UPDRS) score of 71. There was no limb apraxia, dystonia, fasciculations, or myoclonus. Levodopa/Carbidopa was started but she remained poorly responsive to dopamine replacement therapy. She became wheelchair bound and dependent 7 years after disease onset. There were no hallucinations, delusions, visual perceptual dysfunction, sleep disorders or behavioral changes. Cranial MRI of this sister (Figure 3F) and PET scan findings (Figure 3G) were unremarkable. Surface EMG showed tremors. Muscle ultrasound studies revealed no fasciculations.

The fourth sibling carrying the same *OPTN* frameshift insertion (III-6) is a female who was 57 years of age during the time of examination and has remained asymptomatic at the time of writing. MRI and FDG-PET imaging were normal (Figure 3E) and there was no evidence of ALS clinically or on neurophysiological testing. Apart from her, further history revealed that the proband's older brother, III-3, presented with behavioral changes at 54 years old suggestive of bvFTD, but was not formally diagnosed. He would jump off a moving jeepney and paid no attention to street signs, crossing the street carelessly. He had delusions, claiming he had a girlfriend abroad. Eventually he had reduced interest in social interaction. He died at age 57. Other family members (I-1, II-1, II-2, II-4), already deceased, had tremors with an unspecified diagnosis. These tremors were termed “kiriw” which described tremulousness or losing control of the hand in local parlance. These affected individuals were occupationally functional but drank alcohol almost daily which worsened the tremors. They did not develop any clinical manifestations of dementia. No blood specimens were collected from these deceased individuals. There is no history of consanguinity in this family.

## Discussion

In this study, we screened the proband along with both affected and unaffected family members for FTD and parkinsonism-related genes using a custom targeted exome sequencing panel. We found a novel *OPTN* frameshift insertion in all three affected family members and one unaffected sibling. Two homozygous carriers demonstrated an FTD phenotype, including bvFTD and nfvPPA, while the heterozygous sibling had a parkinsonism/CBS syndrome. None of the tested family members without the mutation had FTD or parkinsonism. All carriers had no evidence of ALS at present, and were negative for mutations in *MAPT, GRN* and C9orf72. Interestingly, one heterozygous carrier remains unaffected at age 57 which is past the age of onset of affected siblings. It remains to be seen whether such heterozygous mutation is pathogenic and associated with parkinsonism. This is the first reported family harboring this novel *OPTN* mutation and it presents without evidence of ALS thus far but with a predominant FTD phenotype. FTLD-TDP cases without motor neuron disease have been reported in association with *OPTN* and *TBK1* (Tank-binding kinase (1) mutations (11). Mutations in *TBK1*werealso excluded in our affected family members. A possibility remains that we cannot rule out the presence of subclinical motor neuron degeneration, or that symptoms of ALS have not yet manifested in these affected persons.

The *OPTN* gene is an ubiquitin-binding protein responsible for cell trafficking leading to NF-kappa-B activation and its mechanism is believed to be a loss of function or haploinsufficiency. *OPTN* is a primary receptor required for the selective autophagy of damaged mitochondria; this process is also regulated via *OPTN* phosphorylation by *TBK1* (31). Deletions, nonsense and frameshift mutations in *OPTN* have been found in either homozygous or heterozygous state, suggesting that complete loss of function or haploinsufficiency of optineurin is enough to cause or contribute to ALS (32). Most *OPTN* mutations are heterozygous, except for seven homozygous mutations: p.D127Rfs*21 (33), p.G291fs* (34), p.K359fs* (34), p.E135X (35), p.S174X (36), p.Q398X (37), and Ex5del (13). Homozygous loss-of-function mutations in *OPTN* were first reported in Japanese families with autosomal recessive ALS (13), while heterozygous *OPTN* mutations may confer risk for or be causative for ALS (33, 38, 39). The proband (III-10) and sibling III-8 were both homozygous carriers of the frameshift insertion. While the proband had an earlier age at disease onset (45 years), sibling III-8 had a similar age at onset (AAO) of 52 years as sibling III-7, who was heterozygous.III-6 is a heterozygous carrier and without any symptoms at age 57. There is limited information on whether homozygous *OPTN* carriers have greater loss-of-function and/or variances in clinical presentation including earlier AAO.

While dominant acting variants in *OPTN* appeared to make a substantial contribution to sporadic ALS, Pottier et al. first reported *OPTN* mutations in FTD, with their findings supporting a more complex mode of inheritance: sporadic individuals with heterozygous *OPTN* mutations who develop symptoms may do so because of compound heterozygote mutations (with the second hit not “qualifying” as a pathogenic variant) or because they also carry a mutation(s) in another gene (11). Pathological examination showed typical FTLD pathology without clinical motor neuron disease in 2 cases carrying *OPTN* mutations, a case of early onset dementia with prominent behavioral symptoms and a male with primary progressive aphasia carrying *OPTN* and *TBK1* double mutation, extending the spectrum of *OPTN* and *TBK1* mutations to pure FTLD (11). To our knowledge, however, our family is the first report of a CBS/parkinsonism phenotype in association with *OPTN* mutations.

Aside from ALS, other forms of parkinsonism including the 4-repeat tauopathies of corticobasal degeneration (CBD) and progressive supranuclear palsy (PSP) remain part of the FTD spectrum of disorders (40–43). At present, the most studied forms of autosomal dominant forms of FTD associated with parkinsonism are those linked to Chromosome 17 i.e., mutations in *MAPT* and *GRN*, followed by hexanucleotide repeat expansions in C9 or f72 (2, 44, 45). As mentioned, none of these mutations were reported in siblings III-7 and III-8, who had an asymmetric parkinsonism suggestive of a corticobasal syndrome. Sibling III-7 manifested with rapidly progressive parkinsonism at 52 years but has not demonstrated features of FTD with serial clinical examination thus far. Parkinsonism in FTD may present before, during or after the development of behavioral or language disturbances (45). These symptoms can precede the cognitive and behavioral symptoms of FTD by several years (46) in up to 27% of patients (47). Studies by Baizabal-Carvallo and Jankovic (46) and Gasca-Salas et al. (47) suggest that there is a high probability that parkinsonism among these individuals may precede future onset of FTD and should be evaluated in the serial follow-up of these patients.

Limitations of this study include the absence of histopathological findings, functional validation of the *OPTN* frameshift mutation and lack of DNA samples from other affected and unaffected siblings from other generations. Another limitation is the lack of RNA availability which would have provided information in comparing expression levels between homozygous and heterozygous mutation carriers. However, this is the first report of a novel frameshift insertion at *OPTN* (Chr 10:13166090 G>GA) p.Lys328GluTer11in 4 members of a family from the Philippineswith FTD-related phenotypes. Affected members harboring homozygous mutation presented with FTD-related phenotypes. It should be noted however, that we cannot confer pathogenicity in the heterozygous cases and that parkinsonism may be unrelated. It remains to be determined if this frameshift mutation acts in a recessive and or dominant fashion or whether it is associated with parkinsonism. As such, further close monitoring and follow up of clinical symptoms will be important. We aim for future histopathologic studies in this family to possibly elucidate disease mechanism, unfortunately now, Filipino practice does not allow tampering of the brain post-mortem.

Overall, we identified a novel *OPTN* frameshift insertion in a family with frontotemporal dementia and parkinsonism/CBS but without clinical and neurophysiological evidence of ALS, expanding the phenotypic spectrum of *OPTN* mutations. The absence of *TBK1* gene mutation in individuals without features of ALS and the other genes commonly implicated in FTD-ALS overlap offer these possibilities: (1) subclinical or, (2) early stage of motor neuron degeneration, (3) presence of other ALS-modifying genes. Given the findings in this family, close follow-up of mutation carriers for the development of new clinical features and wider investigation of additional family members with collection of DNA samples and further genetic analyses will be conducted to investigate the possibility of multiple interacting genetic variants/genetic modifier sin this family which could explain the heterogenous disease phenotype.

## Data Availability Statement

Patient WES (whole-exome sequencing) data are deposited in the European Genome-phenome Archive (EGA, https://ega-archive.org, study accession number EGAS00001005220) and are available upon request to Data Access Committee (DAC).

## Ethics Statement

The studies involving human participants were reviewed and approved by St. Luke's Medical Center. The patients/participants provided their written informed consent to participate in this study.

## Author Contributions

JD, JTY, AN, MFD, BN, JC, JY, and MLD contributed to the acquisition, interpretation of clinical, and imaging data. YT, ML, LZ, WL, JF, and ASLN contributed to the collection and processing and analysis of the genetic samples. JD, JTY, and ASLN prepared the initial paper which was reviewed and revised and approved by all authors. All authors contributed to the article and approved the submitted version.

## Conflict of Interest

The authors declare that the research was conducted in the absence of any commercial or financial relationships that could be construed as a potential conflict of interest.

## Abbreviations

ALS: Amyotrophic Lateral Sclerosis
bvFTD: behavioral variant FTD
CBS: corticobasal syndrome
CDR: Clinical Dementia Rating
C9orf72: chromosome 9 open reading frame 72- SMCR8complex subunit
EMG: electromyography
FTD: Frontotemporal dementia
fFTD: familial frontotemporal dementia
MAPT: microtubule associated protein tau
MMSE: Mini Mental State Exam
MoCA-P: Montreal Cognitive Assessment- Philippines
MRI: magnetic resonance imaging
nfvPPA: non-fluent primary progressive aphasia
OPTN: optineurin
GRN: granulin precursor
svPPA: semantic variant primary progressive aphasia
sALS: sporadic ALS
TBK1: tank-binding kinase 1
UPDRS: Unified Parkinson's Disease Rating Scale.
