# Supplementary material for: Novel Optineurin Frameshift Insertion in a Family With Frontotemporal Dementia and Parkinsonism Without Amyotrophic Lateral Sclerosis
Source: Front Neurol. 2021 May 19;12:645913. doi: 10.3389/fneur.2021.645913 (PMC8170397; doi:10.3389/fneur.2021.645913)
Supplement: Supplementary file 1 [file Data_Sheet_1.DOCX]

**Supplementary Materials and Methods**

**C9orf72 genotyping**

GGGGCC repeat sequences in the *C9orf72* gene were amplified using polymerase chain reaction (PCR). The forward and reverse primers were located at the flanking GGGGCC repeat region. The PCR mix contained 0.75 U Taq DNA Polymerase, 10× Taq Buffer, 300 μM each dATP, dTTP, dCTP, 75 μM dGTP and 225 μM 7-Deaza-2′-deoxyguanosine-5′-triphosphate (Roche), 5% dimethyl sulfoxide (Sigma-Aldrich), 2.5 Mm MgCl2, 1 M betaine (Sigma-Aldrich), 0.4 μM each primer (F1 and R1 in Supplementary Table 2) and 50-100 ng genomic DNA in a total reaction volume of 25 μl. After initial denaturation at 96 °C for 3 min, the cycling conditions were as follows: 7 cycles of 96 °C for 30 s, 70 °C for 45 s with a reduction of 2°C per cycle, and 72 °C for 1.5 min, and 28 cycles of 96 °C for 30 s, 56 °C for 45 s and 72 °C for 1.5 min, followed by a final elongation step of 72 °C for 10 min. Electrophoresis was performed on a 3500xl Genetic analyzer with GeneScan 500 Rox dye Size Standard (Applied Biosystems) and the data were analyzed using GeneMapper software (Applied Biosystems). GGGGCC repeats numbers were calculated from the molecular weight of PCR product. For samples that displayed a single peak, repeat-primed PCR was carried out to detect any large GGGGCC repeat expansions. With the same PCR conditions, detection was carried out with primer mix (F2, P3R and P4). Primer P4 contained (GGCCCC)_3_ as a complementary sequence with GGGGCC. Repeat expansions produce a characteristic sawtooth pattern with a 6-bp periodicity.

**Sanger sequencing**

After amplification with exTEN 2x PCR Master Mix (1^st^ Base), PCR products were treated with FastAP Thermosensitive Alkaline Phosphatase (Thermo Scientific) and Exonuclease I (Thermo Scientific) and subjected to sequencing following standard protocol from BigDye® Terminator v3.1 Cycle Sequencing Kit (Applied Biosystems). The amplified products were purified by magnetic beads clean up kit before loading into ABI 3730xl DNA Analyzer for DNA sequencing.

**Whole exome sequencing (WES)**

Whole exome was captured with the NimbleGen SeqCap EZ Human Exome v3.0 (Roche) following the manufacturer’s protocol, and sequenced using HiSeq4000 with 150-bp paired end reads (Illumina). The same variant calling and annotation pipeline, as well as variant filtering criteria utilised for targeted exome sequencing reads was also conducted for the whole exome sequencing data.

**G -> GA insertion at Lys328**

328 329 330 331 332 333 334 335 336 337 338 339 340

Lys Lys Arg Leu Gln Glu Lys Cys Gln Ala Leu Glu Arg

AAG AAG AGA CTT CAA GAA AAG TGT CAG GCC CTT GAA AGG

328 329 330 331 332 333 334 335 336 337 338 339 340

Lys Lys Glu Thr Ser Arg Lys Val Ser Gly Pro Ter

AAG **A**AA GAG ACT TCA AGA AAA GTG TCA GGC CCT TGA AAG G

**Table 1. List of 200 neurodegenerative disease-related genes**

| **Alzheimer’s disease, Frontotemporal dementia, vascular dementia and inherited vasculopathy-related genes (n=127)** | | | | | | | | | |
| --- | --- | --- | --- | --- | --- | --- | --- | --- | --- |
| ABCA7 | ABCD1 | ACE | ACOX1 | ADAM10 | APOC1 | APOE | APP | ARSA | ARID5B |
| ASPA | KCTD2 | AUH | ATXN2 | BIN1 | C11orf30 | C9ORF72 | CALHM1 | CASS4 | CD2AP |
| CD33 | CELF1 | CHCHD10 | CHMP2B | CHRNA9 | CLU | CNTNAP2 | COL4A2 | COL4A1 | CR1 |
| CTC1 | CSF1R | CYP11B2 | CYP19A1 | CYP27A1 | CYP2D6B | CYP2J2 | DARS2 | DNMT1 | DSG2 |
| DYRK1A | DYSF | ECHDC3 | EIF2B1 | EIF2B2 | EIF2B3 | EIF2B4 | EIF2B5 | EPHA1 | ERCC6 |
| ERCC8 | EPHA4 | FBF1 | FGB | FERMT2 | FUS | GAB2 | GALC | GBE1 | GCDH |
| GFAP | GLA | GRN | GSK3B | HEXA | HLA-DRA | HLA-DRB1 | HLA-DRB5 | HS3ST1 | HTRA1 |
| IL23R | INPP5D | L2HGDH | LMNB1 | LRRTM3 | MADD | MAN2B1 | MAPT | MEF2C | MLC1 |
| MRPL38 | MS4A4A | MTHFR | NCAM2 | NEDD9 | NLRP3 | NME8 | NOTCH3 | NXPH1 | NYAP1 |
| OPTN | SERPINE1 | PAXIP1 | PICALM | PLP1 | PLD3 | PRNP | PSAP | PSEN1 | PSEN2 |
| PTK2B | RAB38 | REST | RIN3 | RUNX1 | SIRT2 | SLC17A5 | SLC2A14 | SORL1 | SQSTM1 |
| TARDBP | TBK1 | TMEM106B | TOMM40 | TP63 | TRIM47 | TRIM65 | TREM2 | TREX1 | TYMP |
| TRIP4 | TYROBP | UNC5C | UPP2 | VCP | WBP2 | ZCWPW1 |  |  |  |
| **PD related genes (n=73)** | | | | | | | | | |
| ACMSD | APOOP2 | ATP13A2 | BCKDK | BST1 | CCDC62 | CHCHD2 | COQ2 | CRAT | DDRGK1 |
| DGKQ | DLG2 | DNAJC13 | DNAJC5 | DNAJC6 | EIF4G1 | FAM186A | FAM47E | FBXO7 | FGF20 |
| GAK | GBA | GCH1 | GPNMB | GUCY1A3 | HIP1R | HLA-DRA | INPP5F | ITGA8 | ITPKB |
| KANSL1 | KRT8P25 | LAMC2 | LAMP3 | LRRK2 | MC1R | MCCC1 | MIR4697 | MMP16 | NMD3 |
| NSF | NUCKS1 | PARK2 | PARK7 | PINK1 | PLEKHM1 | PM20D1 | RAB25 | RAB7A | RAB29 |
| RAB7B | RAI1 | RIT2 | SCARB2 | SIPA1L2 | SLC2A13 | SLC41A1 | SLC45A3 | SLC7A4 | SMPD1 |
| SNCA | SPPL2B | SREBF1 | STBD1 | STK39 | STX1B | SYT11 | TMEM163 | TMEM175 | TMEM229B |
| USP25 | VPS13C | VPS35 |  |  |  |  |  |  |  |

200 neurodegenerative disease-related genes were selected for a custom commercial exome panel, which included genes selected from GWAS studies, risk variants as well as causal genes related to Alzheimer’s disease, Frontotemporal dementia, inherited vasculopathies as well as Parkinson’s disease.

**Table 2. Primers used in Sanger sequencing and *C9orf72* genotyping.**

| Target | Sequence |  |
| --- | --- | --- |
| C9orf72 | F1 | 5’ FAM-CAAGGAGGGAAACAACCGCAGCC-3’ |
|  | R1 | 5’-GCAGGCACCGCAACCGCAG-3’ |
|  | F2 | 5’ FAM-TGTAAAACGACGGCCAGTCAAGGAGGGAAACAACCGCAGCC-3’ |
|  | P3R | 5’-CAGGAAACAGCTATGACC-3’ |
|  | P4 | 5’- CAGGAAACAGCTATGACCGGGCCCGCCCCGACCACGCCCCGGCCCC GGCCCCGG-3’ |
| OPTN | F | 5’- TCCCAGTGCATCCAAATTGA-3’ |
|  | R | 5’- AAGTTCTCCAGTCCCCAACC-3’ |
